# Data linkage in social care: a pilot project

**DOI:** 10.23889/ijpds.v1i1.286

**Published:** 2017-04-19

**Authors:** Alison Orrell, Martin Heaven, Diane Seddon, Catherine Robinson

**Affiliations:** Bangor University; Swansea University

## Objectives

To build the complete picture of service provision there is a need to broaden the linked data available to include health, social service provision by Local Authorities, and provision of support by third sector organisations. Data Linkage in Social Care is a pilot project to test the feasibility of linking datasets from a local authority, the NHS and third sector organisations. The focus of this work is individual level data from adults who are referred to social services in order to avoid admission to hospital or to facilitate their discharge from hospital. The data linkage will include data from statutory and third sector organisations and services which provide interventions and support in community settings.

## Objectives

The main aims of this research are to:

1. Test the feasibility of linking datasets from Local Authority, the NHS and third sector organisations.
2. Build a more complete picture of service provision using adults who have been referred to social services in order to avoid admission to hospital or to facilitate their discharge from hospital.
3. Assess the range and quality of data available in each of the relevant organisations providing services to those individuals.

The research outcome is a better understanding of the utility of data linkage across statutory and third party organisations.

## Approach

A Bangor University led research team partnered with the Gwynedd Local authority to explore the Governance Issues and practicalities of providing an anonymised dataset to the SAIL databank at Swansea. Two third sector agencies were also approached. With the various required Service Level Agreements in place, data were put through the tried and trusted SAIL process for analysis. 

## Results

Data relating to well over 20,000 referrals generated by 17,000+ social services clients in Gwynedd Local Authority from the period 2008 to 2015 were anonymised into the SAIL databank in Swansea, and linked to records from primary and secondary care. We will present results on the success of this process and on the emerging findings from the linked datasets. 

